# Nuclear Factor 90(NF90) targeted to TAR RNA inhibits transcriptional activation of HIV-1

**DOI:** 10.1186/1742-4690-4-41

**Published:** 2007-06-12

**Authors:** Emmanuel T Agbottah, Christine Traviss, James McArdle, Sambhav Karki, Georges C St Laurent, Ajit Kumar

**Affiliations:** 1 Department of Biochemistry & Molecular Biology, School of Medicine, The George Washington University, Washington D.C. USA

## Abstract

**Background:**

Examination of host cell-based inhibitors of HIV-1 transcription may be important for attenuating viral replication. We describe properties of a cellular double-stranded RNA binding protein with intrinsic affinity for HIV-1 TAR RNA that interferes with Tat/TAR interaction and inhibits viral gene expression.

**Results:**

Utilizing TAR affinity fractionation, North-Western blotting, and mobility-shift assays, we show that the C-terminal variant of nuclear factor 90 (NF90ctv) with strong affinity for the TAR RNA, competes with Tat/TAR interaction *in vitro*. Analysis of the effect of NF90ctv-TAR RNA interaction *in vivo *showed significant inhibition of Tat-transactivation of HIV-1 LTR in cells expressing NF90ctv, as well as changes in histone H3 lysine-4 and lysine-9 methylation of HIV chromatin that are consistent with the epigenetic changes in transcriptionally repressed gene.

**Conclusion:**

Structural integrity of the TAR element is crucial in HIV-1 gene expression. Our results show that perturbation Tat/TAR RNA interaction by the dsRNA binding protein is sufficient to inhibit transcriptional activation of HIV-1.

## Background

Highly Active Antiretroviral Therapy (HAART) administration utilizes a combination of inhibitors of viral protease and reverse transcriptase to markedly reduce circulating viral levels [1,2]. However, the emergence of drug-resistant variants eventually limits the benefits of chemotherapy; hence the need for alternate or complementary strategies.

The nascent transcripts from HIV-1 Long Terminal Repeat (LTR) contain a unique structured RNA domain within the 5'-nontranslated region known as the transactivation response (TAR) element which is critical for efficient transcription of viral promoter in response to Tat [3,4]. The TAR RNA element extends between nucleotides +1 and +59 and forms a stable RNA stem-loop structure [5,6]. Studies on the transactivation mechanism involving the Tat-TAR interaction have yielded significant insights into the regulation of viral gene expression [7-10]. The primary role of Tat may in fact be to promote assembly of pre-initiation complex, thereby promoting both transcription initiation and elongation of HIV-1 promoter [4]. It is likely therefore, that Tat facilitates chromatin modifications, thereby promoting initiation and transcription elongation in a series of sequential, coordinated events that lead to high levels of HIV transcription [11]. Consistent with this view, we noted that Tat/TAR-specified CDK9 (P-TEFb) kinase activity is critical for the phosphorylation of RNAP II, transcription elongation factors SPT5 and Tat-SF1 and the induction histone H3 lysine 4 and lysine 36 methylations during transcriptional activation of integrated HIV-1 chromatin [12]. We reasoned therefore that competition of Tat/TAR interaction by dsRNA binding protein, such as NF90ctv, might interfere with viral gene expression *in vivo*. Given the functional importance of Tat-TAR interaction in viral life cycle; Tat protein and the TAR element both present attractive targets for therapeutic drug design.

Agents affecting the Tat/TAR interaction could prevent transcriptional activation of HIV-1 genome either by steric hindrance, a shear displacement mechanism, or by deprivation of Tat-cofactor molecules (i.e. CBP/300, Tat-SF1) [13,14]. The inhibitors of Tat/TAR axis include TAR RNA decoys [15,16], small molecule inhibitors and ribozyme [17-24]. Other Tat inhibitors that directly compete with Tat function include anti-Tat monoclonal antibody and single-chain anti-Tat antibodies [25-29].

NF90ctv is a C-terminal variant [30] of the NF90 double-stranded RNA (dsRNA)-binding protein which was originally reported as a putative transcription factor recognizing the antigen receptor response element (ARE) in the IL-2 gene regulatory region [31]. A shared feature of the dsRNA binding proteins is their conserved N-terminal domains and the C-terminal dsRNA binding motifs [32]. This motif is well conserved through evolution and interacts with dsRNAs as well as structured RNAs such as the adenovirus VA RNA II [33]. NF90 has two dsRNA binding motifs, a putative nuclear localization signal (NLS), and a leucine-rich nuclear export signal (NES). The C-terminal portion of NF90 contains an arginine, glycine-rich (RGG) domain, similar to the motifs which mediate RNA binding by hnRNP-U and nucleolin [34].

We studied the unique C-terminal variant of NF90 (NF90ctv), where the C-terminal 70 amino acids of arginine/glycine rich domain is substituted largely by acidic residues due to a CT insertion in exon 15 that alters the translational reading frame. Cells expressing NF90ctv stimulate a transcriptional program of IFN response genes which is responsible in part for their ability to inhibit HIV-1 replication [30]. NF90ctv (670a.a) differs from the related proteins, NF90a (702a.a) and NF90b (706a.a). Mathews and colleagues analyzed the dsRNA binding properties of NF90 family of proteins and suggest that NF90ctv displays ten fold higher affinity for dsRNA as compared with the normal C-terminal domain RG-rich proteins of NF90 family [33]. We examined the TAR RNA binding properties of NF90ctv and show that it attenuates HIV-1 replication in part by inhibition of Tat-mediated transactivation of HIV-1 LTR.

### Experimental procedures

#### Plasmids

Recombinant plasmids for expression in mammalian cells were constructed as follows: pJK2 (HIV-1LTR/β-galactosidase reporter), pSV_2_-Tat72, (SV40 promoter driven vector encoding the 72 amino acid first exon of Tat), pCMV-NF90ctv (CMV promoter driven construct of original NF90ctv expression vector was supplied by Dr. Peter Kao, Stanford University CA) [31]. pOZ (bicistronic vector) and pOZNF90ctv (POZ vector expressing NF90ctv used in stable cell creation as described below).

#### Tissue culture and HIV-1 infection

GHOST(3)CXCR4 cells were obtained from the NIH AIDS Research and Reference Program. The cell line is derived from human osteosarcoma (HOS) cells by stable transduction with HIV-2 long terminal repeat (LTR)-driven green fluorescent protein (GFP) reporter, human CD4 receptor, and human CXCR4 chemokine receptor genes. To generate cell lines stably expressing NF90ctv, GHOST(3)CXCR4 cells were transduced with pOZNF90ctv or the plasmid with 'empty vector', using transduction and selection protocols described elsewhere [30]. For HIV-1 infection, T-tropic HIV-1 strain NL4-3 was obtained from the NIH AIDS Research and Reference Program. Virus infection was performed by incubating the cells with HIV-1 NL4-3 (at approximately 5 ng of p24 gag antigen per 10^6 ^cells) in 0.5 ml culture medium supplemented with 20 μg/ml polybrene. An aliquot of the culture supernatant was collected and stored at -80°C. Production of p24 antigen was analyzed by enzyme-linked immunosorbent assay (ELISA) according to the manufacturer's instructions (Beckman Coulter, Fullerton CA.).

#### Purification and characterization of TAR binding protein NF90

The purification and characterization of NF90ctv binding to TAR RNA was examined by stepwise fractionation of HeLa nuclear extract. The SP Sepharose chromatography fractions eluted at 0.25 M and 0.5 M NaCl were applied to a TAR affinity column. Briefly, 200–250 μg of biotinylated TAR RNA was bound to NutrAvidin Plus beads (Pierce, Rockville, IL). Binding of RNA to affinity beads packed in a 0.8 × 7.0 cm column occurred for 30 minutes at 4°C with rocking. The SP Sepharose pool was applied to the column, recycled four times and the TAR RNA bound fractions were recovered with a 2.0 M KCl step elution. Proteins contained in the partially purified TAR fraction were analyzed by SDS-PAGE and North-Western blots [37].

**Electrophoretic mobility shift **assays (EMSA) with labeled TAR RNA revealed that the SP Sepharose and the TAR RNA bound fractions were able to retard TAR RNA mobility in non-denaturing acrylamide gels.

#### Competition analysis and RNA binding specificity

500 ng of protein from the TAR RNA purification step was incubated with 0.2 pmoles of radiolabeled TAR RNA. Competition for radiolabeled TAR RNA binding was done with increasing amounts of unlabeled wild-type TAR RNA and mutant TAR RNA transcripts TM12, TM18 and TM27.

#### NorthWestern analysis

Equal amounts of protein (25 μg) from the TAR RNA bound fraction was transferred to immobilon-P and blocked for 2 hours in NorthWestern buffer (20 mM HEPES pH 7.9, 50 m M KCl, 0.5 mM EDTA, 2 mM MgCl_2_, 0.01% NP40 containing 5% milk). Following a 10 minute wash, the bound protein fraction was probed with ^32^P-labeled TAR RNA (5 × 10^5 ^cpm/ml, 400 ng) in the NorthWestern buffer containing 0.2% milk and 50 U/mL RNasin for 2 hours. The blots were washed, air dried and exposed for autoradiography overnight at -70°C. Control RNA probe included human globin RNA. TAR RNA bound protein fractions (25 μg) were also monitored by Western blot analysis and probed with anti-NF90 polyclonal antibody.

#### Transiently transfected HeLa and Jurkat cells

The effect of NF90ctv on Tat-mediated transactivation of HIV-1 LTR was assessed in both HeLa and Jurkat cells using CaCl_2_/HEPES transfection procedure. Constant (2.5 μg) amount of pHIV-LRT-β gal reporter (LacZ gene under the control of the HIV-1 LTR) was cotransfected with increasing amounts of pCMV-NF90ctv (0, 2, 10 μg), or together with 5 μg of pSV_2_-Tat72 encoding the 72 amino acid first exon of Tat. To control for transfection efficiency, 0.2 μg of pCMV-CAT plasmid DNA was cotransfected. The final total amount of DNA in each reaction was adjusted with salmon sperm DNA. After 48 hours, cell extracts were prepared and standardized for total protein using a modified Bradford assay (Bio-Rad). Colorimetric β-galactosidase and CAT assays were performed as described [37]. In each case β-galactosidase reporter was normalized to protein concentration based on CAT values used as transfection control.

#### Northern blot

Total RNA was isolated from cells using RNAZOL (TEL-TEST, TX USA). Briefly, 20 × 10^6 ^of non-infected or HIV-1pNL4-3 or pseudotyped VSVG-HIV-1 infected GHOST-CXCR4/pOZ-NF90 or GHOST-CXCR4/pOZ empty-vector transduced cells were washed twice with PBS and lysed in culture flask by addition of 5 ml of RNAZOL. Following extraction with 0.5 ml chloroform, the RNA was precipitated with isopropanol and washed with 75% Ethanol. 15 μg of total RNA were loaded into each lane of 1% Formaldehyde-agarose gel and electrophoresed under standard conditions. The RNA was transferred to Nitrocellulose membrane (Schleider & Schuell, Keene, NH) by capillary action using 10 × SSC and cross-linked using ultraviolet light. Membranes were prehydrated in 6 × SSC, 1% SDS solution containing 150 μg of salmon sperm DNA for 2 hours at 65°C. The pre-incubated blots were hybridized at 65°C in shaking water bath for approximately 20 hours with ^32^P-random prime labeled DNA fragment of whole HIV genome (Lofstrand, Gaithersburg, MD). Membranes were washed twice (5 minutes each in 1 × SSC 1% SDS at room temperature), at 15 minutes each in 1 × SSC, 10% SDS at 37°C and finally for 1 hour at 0.1 × SSC 1% SDS at 65°C. The blots were wrapped in Saran wrap and the radioactive bands were detected (Molecular Dynamics, Sunnyvale, CA). To control for RNA loading, levels of 18S and 28S or β-actin RNA were used as reference.

#### Competition of TAR/TAR complex with NF90c protein

One microgram of purified biotin-labeled TAR RNA was mixed with one microgram (1 μg) of purified Tat protein for 10 minutes on ice. Next, 100 μl of 30% strepavidin-sepharose beads in binding buffer (50 mM Tris-HCl, pH  7.8; 5 mM DTT, 100 μg of BSA, 60 mM KCl and 5 mM MgCl_2_) were added to the reaction for a final volume of 200 μl. The TAR/Tat complex was incubated with beads for an additional 1 hr on ice. Next, various concentrations of purified NF90ctv protein (0.1, 1, and 5 μg) were added to the mixture. All samples were further incubated on ice for an additional hour. Finally, samples were centrifuged at 4°c for 5 minutes, and washed (3X) with TNE_300 _+ 0.1% NP-40 (50 mM Tris-HCl, pH  7.8, 300 mM NaCl, 1 mM ETDA, plus 0.1% NP-40). A final wash with TNE_50 _+ 0.1% NP-40 was performed. Bound complexes were separated on a 4–20% SDS/PAGE and Western blotted either with anti-Tat mAb or anti-NF90c antibodies. The same Blot was cut in half for either Tat or NF90c Western blot.

#### ChIP assays in OM10.1 cells

OM10.1 cells, a promyelocytic line containing transcriptionally latent, single copy of wild-type HIV-1 integrated proviral DNA (subtype B, LAI strain) [38], were induced with TNF-α, either without or following transfection with the NF90ctv expression plasmid. Approximately 5 × 10^7 ^OM10.1 cells were induced with TNF-α (10 ng/ml) for 2 hrs and cross-linked (1% formaldehyde, 10 min at 37°C), and samples were sonicated to reduce DNA fragments to ~200 to 800 bp for ChIP assays essentially as described earlier [12]. DNA bound proteins were immunoprecipitated with approximately 10 μg of antibodies indicated in the figure legends. Specific DNA sequences in the immunoprecipitates were detected by PCR using primers specific for HIV-1 LTR (-92 to +180) and Env (+8440 to +8791) regions.

## Results

### Isolation and identification of HIV-1 TAR binding proteins

**Figure 1:**
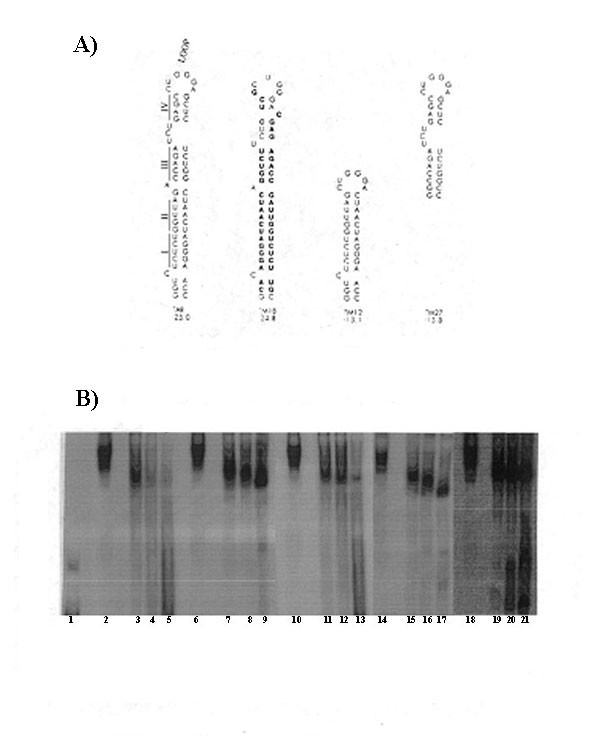
**Competition Analysis of RNA Binding Specificity. A: **The structure of the wild type TAR RNA and the TAR mutants used in this assay are illustrated. **B: **500 ng of protein from the TAR Fraction containing NF90 was incubated with 0.2 pmole radiolabeled TAR RNA (lanes 2, 6, 10, 14, 18). Competition for radiolabeled TAR RNA binding was done with increasing amounts of unlabeled TAR RNA (lanes 3, 5), TM12 RNA (lanes 7, 9), TM18 RNA (lanes 11–13), TM27 RNA (lanes 15–17), or TM12+TM27 RNAs (lanes 19–21). Samples were run on a 10%PAGE.

To detect proteins that interact with the TAR region of HIV-1 LTR, a three-step purification process was devised to fractionate nuclear proteins from HeLa cells, including a Sulpho-phosphate Sepharose (SP Sepharose) chromatography step followed by TAR RNA affinity chromatography. The final fraction, a 2.0 M KCl eluate (TAR affinity fraction) contained proteins p160, p110, p90, and p62. To determine which proteins from the TAR affinity fraction directly interacted with TAR RNA, North-Western analysis was performed using radio-labeled TAR RNA, with beta-globin RNA as a control. The identity of the 90 kDa band was confirmed by Western blotting using anti-NF90 polyclonal antibody and further established through N- terminal sequence analysis. As described (Figure 1), specificity of NF90ctv binding to the TAR RNA was assessed using selected TAR RNA mutants (TM12, TM18 and TM27; Figure 1A), non- specific dsRNA, poly-IC (data not shown), and by competition reactions (with unlabeled TAR RNA mutants incubated with radiolabeled wild-type TAR RNA; Figure 1B).

**Figure 2:**
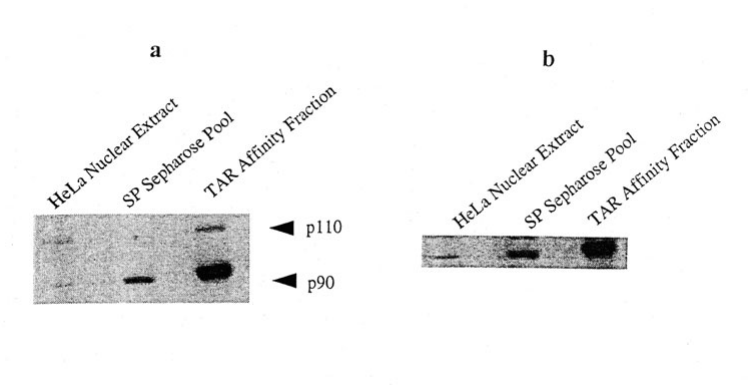
**Identification of NF90 as HIV-1TAR binding protein. a: **Autoradiogram of a NorthWestern blot in which 25ug of HeLa cell nuclear protein from each purification step was probed with 7.5 × 10^6^ cpm of radiolabeled TAR RNA for 2 hours, washed, and exposed to autoradiographic film for 2 hours. **b: **Immobilon-P transferred proteins were probed with polyclonal anti-NF90 at 1 ug/mL.

Figure 2a shows two proteins, 110 kDa and 90 kDa, that specifically recognized by TAR RNA in North-Western blots. As controls we utilized human beta-globin RNA probes in North-Western blotting that showed no p110 or p90 bands (data not shown). The results suggested specific affinity of p110 and p90 to the TAR RNA. We used equal amounts (25 μg) of protein from each of the purification steps in the North-Western assay that were probed with radio-labeled TAR RNA (Figure 2b). Based on the fact that a similar protein input from each fractionation step was used for binding to TAR RNA probe, we estimated (as judged by densitometer analysis, Figure 2a), that the intensity of TAR RNA recognition for p90 was approximately 20-fold higher than the recognition of p110. The p110 protein is an alternatively spliced form of a family of double stranded RNA (dsRNA) binding proteins that includes nuclear factor 90 (NF90) [32]. Two isoforms of p110 have been identified, NF110a (894a.a) and NF110b (898a.a). These dsRNA binding proteins are identical at their N-terminus and have distinct C termini as a result of alternate splicing [32,33]. The enrichment of the 90 kDa protein bound to TAR RNA was further ascertained by Western blotting with NF90 polyclonal antibody (Figure 2b). Sequencing of the N-terminal amino acids of 90 kDa protein and comparison with the protein data bank confirmed its identity to NF90 sequence reported by Corthesy and Kao [31]. All subsequent assays of the TAR RNA binding were carried out with NF90 protein expressed with the cDNA vector (courtesy of Dr. Peter Kao).

### Determination of NF90 binding site on the TAR RNA

As the interaction of proteins with the TAR RNA is likely to be dependent on the RNA structure, in addition to the recognition of the linear sequence motifs, we investigated the possible binding site of NF90ctv to TAR RNA using structural mutants of TAR RNA, and competition with unlabeled RNAs. The secondary structures and free energies of the mutant TAR RNA were predicted with an RNA-folding program [39]. The TAR RNA mutants included base substitutions and deletions of the TAR domain as indicated in Figure 1A. Competition reactions were carried out with increasing concentrations of unlabeled mutant TAR RNAs in the presence of constant amount of radio-labeled "wild type" TAR RNA. The competition of TAR RNA-NF90ctv protein complex was assessed on the basis of the ribonucleoprotein (RNP) complexes formed in the gel mobility shift assays, utilizing non-denaturing polyacrylamide gel electrophoresis.

The TAR RNA mutant, TM18, represents an "antisense" TAR with major alterations in the primary sequence of stem I, II, III, and IV, while retaining the secondary structure of wild type TAR RNA (Figure 1A). When TM18 was used to compete with the RNP complex, free labeled TAR RNA probe was released at 50 pmols, whereas at 10 pmols of unlabeled TAR TM18 there is only a partial competition (Figure 1B lanes 11–13). Thus, NF90ctv appears to have an affinity for the double stranded stem-loop structure of the RNA target that is not dependent on the primary sequence.

To determine whether full length TAR RNA was required for NF90ctv binding or segments of the TAR RNA structure are sufficient, we utilized deletion mutants of HIV-1 LTR. The TAR mutant TM12 contained the lower TAR stem regions I and II and the loop sequence from the wild type TAR (Figure 1A). Results showed that TM12 was not able to compete with wild-type TAR RNA binding to NF90 (Figure 1B lanes 7–9). The TAR mutant, TM27, representing the upper stem regions III and IV and the loop domain of the wild type TAR RNA was partially able to compete TAR RNA binding to NF90ctv (Figure 1B lanes 15–17). Results of the competition experiment that utilized both TM12 and TM27 TAR mutants suggested that the 'two halves' of TAR RNA were not able to compete for NF90 binding (lanes 19–21; compare each competition using mutant TAR RNAs by the competition with wild-type TAR RNA, lanes 3–5). These observations suggest that binding to NF90ctv to HIV-1 TAR RNA requires full length TAR RNA structure with only partial dependence on primary sequence (compare lanes 3–5, showing competition with the wild-type TAR RNA, and lanes 11–13 showing competition with the 'antisense' mutant TAR RNA, TM18).

### Inhibition of Tat-mediated transactivation of HIV-1 LTR by NF90

**Figure 3:**
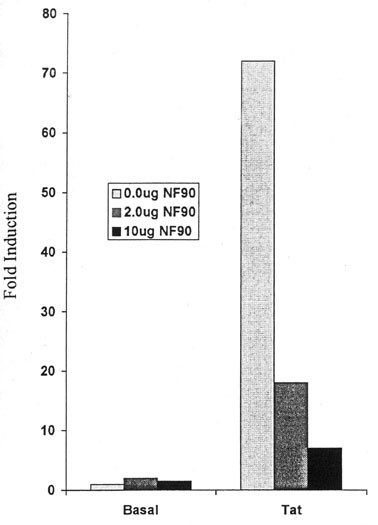
**NF90 inhibits Tat- trans-activation in concentration-dependent manner**. The effect of NF90ctv on basal and Tat-mediated transactivation of HIV-1 LTR was assessed in both HeLa and Jurkat cells using CaCl_2_/Hepes transfection as follow. Duplicate 6 well- plates of HeLa cells received 2.5 ug of pHIV-β gal per 5 × 10^6 ^cells and increasing amounts (0.0 ug, 2.0 ug or 10.0 ug) of pCMV-NF90ctv. One set of plate (Transactivation received constant amounts (5.0 ug) of pSV_2_-Tat72. In each assay, 0.2 μg of pCMVCAT was cotransfected to monitor transfection efficiency. Following the transfection, cell extracts were prepared and standardized for total protein; colorimetric β-galactosidase and CAT assays were performed as before [37).

We initially examined the effect of NF90ctv on basal (Tat-independent) HIV-1 transcription in HeLa cells by transfecting (2.5 μg) of pHIV-LTR/β-galactosidase reporter plasmid, and increasing amounts (0.0, 2, or 10.0 μg) of pCMV-NF90ctv per 5 × 10^6 ^cells using CaCl_2_/Hepes precipitation method (Figure 3, left panel). The effect of NF90ctv on transcription activation was analyzed by adding constant amount (5 μg) of pSV_2_-Tat72 and increasing amounts of NF90ctv plasmid (Figure 3, right panel). Transfection efficiency was normalized by co-transfection with pCMVCAT. In each case the total amount of DNA transfected was kept constant by addition of sonicated salmon sperm DNA. Results indicate that NF90ctv does not exert a noticeable effect on basal (Tat-independent) transcription levels (Fig. 3, left panel). Cells co-transfected with NF90ctv and pSV_2_-Tat72 displayed a significant decrease in Tat-transactivation levels. Cells that received only the Tat construct displayed over 70-fold induction of the β-galactosidase reporter. Tat transactivation was significantly reduced by increasing NF90ctv expression in HeLa cells (Figure 3, right panel).

### NF90ctv reduces HIV-1 RNA levels

**Figure 4:**
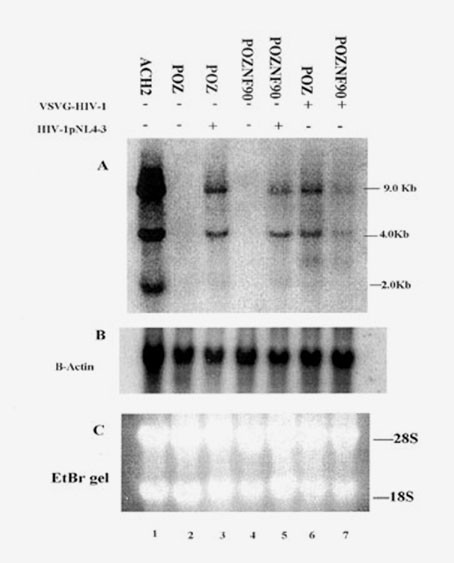
**NF90ctv impacts HIV-1 replication at the transcription level**. Total cell RNA extracted from NF90ctv expressing cells (pOZNF90, lanes 4,5) or control cells transduced with empty vector (pOZ, lanes 2,3) infected with HIV-1pNL4-3 or pseudotyped VSVG-HIV-1 for single round infection (lanes 6,7), was fractionated on a 1% forlmadehyde-agarose gel and probed with [32p]-labeled HIV-1LTR probe. Shown in Figure 4A is a Northern blot of HIV-1 p NL4-3 and VSVG-HIV infected and non-infected cells (designated as plus (+) or minus (-) respectively. Lane 1 is positive control HIV RNA from ACH2 cells. Figure 4B shows the same gel probed with β-actin. Figure 4C illustrates the ethidium bromide-stained gel shown in Figure 4A and 4B.

To assess whether NF90ctv affects HIV-1 transcripts *in vivo*, we analyzed RNA isolated from HIV-1 infected cells that were stably transduced with NF90ctv expressing vector as compared with control cells transduced with the empty vector [30]. Northern blot assays were carried out on total cell RNA isolated from the GHOST-CXCR-4 cells infected either with HIV-1 PNL4-3 or HIV-1 pseudotyped with vesicular stomatitis virus G protein (HIV-VSVG) to analyze the effect of NF90ctv in single-round infection. NF90ctv expression was monitored by Western blot using both polyclonal anti-NF90 antibody as well as anti-FLAG monoclonal antibody. Virus production was monitored by measurement of p24 levels in the culture media by enzyme-linked immunosorbent assay (ELISA) (Beckman-Coulter, California). Twenty micrograms (20 μg) of total cell RNA was resolved on a 1% agarose-formaldehyde gel and probed with 32p-labeled HIV-1 probe. Comparison of lanes 3 and 5 in Fig. 4 shows NF90ctv inhibition of viral transcripts in cells infected with T-tropic HIV-1 NL4-3. The level of inhibition of the 9.0 Kb full length RNA was higher (5 fold) than that of the 4.0 Kb (3 fold), partially spliced transcripts. The doubly spliced, 2 kb RNA transcripts appeared to be minimally represented in both cells. The results could also be explained if NF90ctv also blocks cellular splicing machinery or promotes mRNA decay. Viral RNA from ACH2 cells (T-cells latently infected with HIV-1 that could be induced with sodium butyrate [40] was used as an internal marker (Fig 4A, lane 1). In the case of single cycle infection with pseudotyped VSVG-HIV, both the 9.0 Kb and the 4.0 Kb transcripts were markedly inhibited in the presence of NF90ctv (about 8-fold inhibition of both the 9.0 kb and 4.0 kb transcripts, Figure 4A lanes 6 and 7). Figure 4B shows Northern blot of the same gel probed with [32p]-labeled β-actin to illustrate the equivalent amount of total RNA in each lane. Ethidium bromide stained gel of the RNA samples is illustrated (Fig 4C).

### NF90ctv competes with Tat for TAR RNA binding

**Figure 5:**
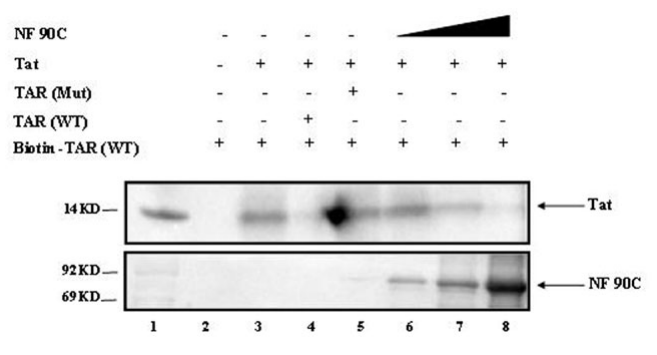
**Competition of TAR/TAR complex formation by NF90c protein**. One microgram of purified biotin-labeled TAR RNA was mixed with one microgram of purified Tat protein for 10 minutes on ice. Next, 100 μl of 30% strepavidin sepharose beads in binding buffer (50 mM Tris-HCl, pH7.8; 5 mM DTT, 100 μg of BSA, 60 mM KCl and 5 mM MgCl_2_) were added to the reaction for a final volume of 200 μl. The TAR/Tat complex was incubated with beads for an additional 1 hr on ice. Next, purified NF90c at various concentrations (0.1, 1, and 5 μg) were added to the mixture. All samples were further incubated on ice for additional one hour. Finally, samples were centrifuged at 4°c for 5 minutes, and washed (3×) with TNE_300 _+ 0.1% NP-40 (50 mM Tris-HCl, pH7.8, 300 mM NaCl, 1 mM ETDA, plus 0.1% NP-40). A final wash was with TNE_50 _+ 0.1% NP-40 was performed. Bound complexes were separated on a 4–20% SDS/PAGE and western blotted with anti-Tat mAb or anti-NF90c antibodies. Same Blot was cut in half for either Tat or NF90c western blot. Lane 1: ^14^C protein molecular weight marker, Lane 2 is with no Tat, Lane 3 with Tat, Lane 4 and 5 are with addition of five microgram of either wild type TAR or a mutant TAR RNA (TM26) as specific and non-specific competitors, respectively. Lanes 6–8 represent addition of purified NF90ctv protein in presence of constant amount of Tat.

We next asked whether NF90ctv could effectively compete with Tat for TAR RNA binding. To do this, we designed a competition experiment where biotinylated wild type TAR RNA was mixed with Tat protein for 10 minutes and then increasing concentration of purified NF90ctv protein was added to the mixture. The resulting complex was then washed and bound proteins were resolved by 4–20% SDS/PAGE, and Western blotted with either anti-Tat or anti-NF90ctv antibody. Results of such an experiment is shown in Figure 5, where wild type Tat specifically bound to TAR RNA and the binding could be competed with excess wild type but not mutant TAR (TM26; compare lanes 4, 5, upper panel). The TAR mutant, TM26 has base substitutions of the Tat-binding pyrimidine bulge region and the cyclin T1 binding loop domain of TAR RNA [36]. However, when increasing concentrations (0.1, 1, 5 μg) of purified NF90ctv protein was added to the complex, Tat-TAR RNA complex was displaced with NF90ctv (Lanes 6–8, upper panel). These results imply that Tat bound to the TAR RNA may be displaced by NF90ctv, thus allowing NF90ctv to interfere with HIV-1 expression.

### Interference of Tat-transactivation by TAR RNA-NF90ctv binding results in histone methylation of HIV chromatin

**Figure 6:**
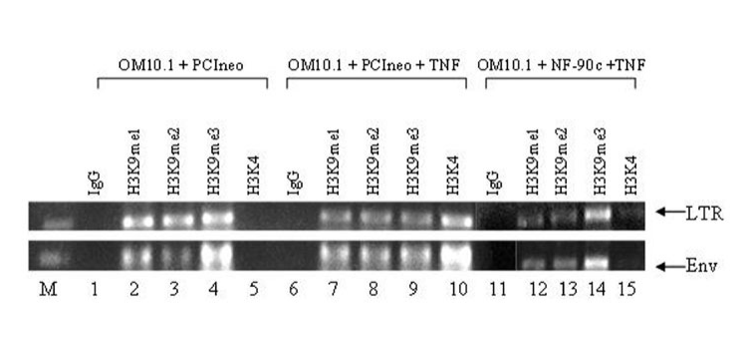
**Competition of Tat/TAR interaction by NF90ctv interferes with HIV-1 chromatin activation: **Changes in Histone H3 K4 and H3K9 methylation were measured by ChIP assays in integrated HIV-1 chromatin in OM10.1 cells. Comparison of H3Kme3 in uninduced (lane, 5) and TNF-α induced cells (lane, 10) shows marks of transcriptional activation of HIV-1 chromatin. In cells expressing NF90ctv however, the H3K4me3 methylation, a mark of transcriptionally active gene is inhibited (lane 15). Lanes 1, 6, and 11 represent IP controls. The empty vector (pCI-neo) was used as transfection control for NF90ctv transfected OM10.1 cells (lanes 11–15). H3K9 methylations (lanes 2–4, 7–9 and 12–14) show a reduction in TNF-α induced cells (compare lanes 2–4 and 7–9). Note the lack of inhibition of H3K9 methylation (lanes 4, 9, and 14) in TNF-α induced cells in the presence of NF90ctv (lane 14), suggesting a long-term memory of transcriptional inhibition in NF90ctv expressing cells.

We previously reported that transcriptional activation of latent proviral DNA by TNF-α induction of OM10.1 cells, a promyelocytic cell line that contains single copy integrated proviral DNA, is mediated by Tat [12]. It was of interest therefore to ask if the interference of Tat-transactivation by NF90ctv would result in inhibition of transcriptional activation of HIV-1 LTR. To approach the issue we analyzed the methylation of histone H3K4 and H3K9 in HIV-1 chromatin by chromatin-immunoprecipitation (ChIP) assays in latent and TNF-α induced OM10.1 cells, and compared the HIV-1 chromatin modification in normal and NF90ctv transfected OM10.1 cells. The results of ChIP assays (Figure 6) make several points: (i) histone H3K4me3 levels (compare Fig.6, lanes 5, 10 and 15) clearly show a marked induction of H3K4me3 upon transcriptional stimulation of latent HIV-1 LTR. However, in NF90ctv transfected OM10.1 cells, there is only minimal induction of H3K4 methylation, even after TNFα induction (compare LTR lanes 10 and 15). Considering that H3K4 methylation marks active genes [41], the results suggest that competition of Tat/TAR RNA interaction by NF90ctv protein results in inhibition of HIV gene expression.

Among the epigenetic marks of transcriptionally silenced chromatin, histone H3K9 methylation, and in particular different degrees of H3K9 methylation suggest regulated suppression of transcriptionally active chromatin [42]. Histone lysine-9 trimethyl (H3K9me3) state in particular, is regarded as a more robust signal of long-term epigenetic memory. We next determined the levels of mono-, di-, or tri-methyl H3K9 in HIV chromatin in the inactive, TNF-α induced and NF90ctv expressing TNFα induced OM10.1 cells. As is shown in Figure 6 (compare lanes 2–4 with lanes 7–9), there is relative decline in H3K9me1, H3K9me2 and H3K9me3 as the HIV chromatin is transcriptionally induced. In TNF-α induced OM10.1 cells which are also transduced with NF90ctv expression plasmid (lanes 12–14), there is a relative induction of H3K9me3 (Fig.6, compare lanes 12, 13 with lane 14). The results suggest that interference of Tat-transactivation of HIV-1 due to the competition of Tat/TAR interaction by NF90ctv results in long-term inhibitory epigenetic memory [42]. The results, however, do not suggest a direct role of NF90ctv in epigenetic modifications of HIV-1 chromatin. Rather, the data suggest an indirect role of NF90ctv-mediated interference of Tat/TAR interaction that leads to the transcriptional repression.

## Discussion

Eukaryotic cells depend on recognizable RNA secondary structures for a variety of normal cellular pathways. Cellular RNA-binding proteins appear to function as information sensors, extracting the information content from specific RNA structural domains to initiate downstream signaling that translate analog to digital information [57]. Significant examples of such RNA structure-directed signaling include viral RNA-mediated activation of innate antiviral defense [reviewed in [54]]. It has been speculated that mammalian cells employ RNA interference (RNAi) pathway as an antiviral mechanism. The HIV-1 TAR RNA binding protein (TRBP) has an essential role in HIV replication as well as in RNAi response where it mediates the association of Dicer with siRNA and Ago2 in RISC complex [reviewed in [55]]. Viruses display clear tissue tropism, suggesting that cellular microRNA (miRNA) regulated genes could modulate viral pathogenesis. Recent studies showing regulation of HIV-1 replication by the miRNA modulated host proteins (which are essential for virus replication), further supports the importance of RNAi-mediated antiviral defense [reviewed in [56]]. Recognition and information extraction for downstream signaling of specific structural domains in RNA may define the outcome of the viral-host interaction.

In the present study we utilized a C-terminal variant of the cellular RNA-binding protein (NF90ctv) that was initially isolated by HIV-1 TAR RNA affinity fractionation. We focused on the C-terminal variant of NF90 to further explore its mechanism of action as a potential antiviral target protein. In an earlier report [30], we described that CD4/CXCR4 positive human osteosarcoma cells stably expressing NF90ctv were able to induce transcriptional program of antiviral response genes to block HIV-1 replication. We recently reported [43] that co-expression of NF90ctv competes with HIV-1 Rev-RRE interaction and may contribute to antiviral response. In the present study we extended the analysis viral RNA-NF90ctv interaction to ask whether NF90ctv binding to HIV TAR RNA competes with Tat-TAR RNA interaction and attenuates of HIV-1 replication. These studies allow us to examine the information 'coded' in the TAR RNA structure, as the basis of recognition by the host protein, and the impact of these interactions on viral replication. Specifically, we used NF90ctv to target sites on the TAR RNA, to disrupt viral processes that require unimpeded access to TAR RNA.

The idea that TAR may be a novel target for drug intervention is supported by the fact that any natural or induced mutation that destabilizes the TAR structure disrupts base pairing in the TAR stem region [44]. Also loss of TAR specific secondary structure abolishes Tat-stimulated transcription resulting in premature transcription termination at random locations downstream of the viral RNA start site [44,45].

NF90 and NF45 are subunits of a heterodimeric protein originally isolated as a putative transcription factor thought to bind to the antigen receptor response elements (ARRE) of the IL-2 gene promoter [31]. Interestingly, subsequent studies have characterized NF90 interactions with RNA species such as the IL2 mRNA [46], the Tau mRNA in neurons [47], and the ADAR1 RNA editing complex [48]. NF90's recognition of these RNA species depends on secondary structure, rather than primary sequence; our studies show that NF90 recognition of RNA species depends on somewhat constrained structural parameters.

NF90 protein has two dsRNA binding domains as well as a putative nuclear localization signal (NLS), a leucine-rich nuclear export signal (NES) and a C-terminal domain that is arginine, glycine (RG) rich [33]. In quiescent Jurkat cells, NF90 is predominantly localized in the nucleus, however, in response to activation signals there is an increase in its cytoplasmic translocation. Increased cytoplasmic abundance of NF90 depends on the presence of its nuclear export sequence (NES) [46]. The NF90 cDNA originally reported [31], and used in this work is a polymorphic C-terminal variant (NF90ctv), which results from a dinucleotide (CT) insertion that results in translation frame-shift giving rise to a protein with a shorter C terminus lacking both the RGG and GQSY domains [35]. As we reported earlier [30], real-time quantitative RT-PCR analysis suggests that the endogenous levels of NF90ctv are undetectable in cell lines we studied; levels of the NF90 C-terminal variants in human primary cells is not known.

Reichman and Mathews [33] analyzed the RNA binding properties of NF90 family of dsRNA binding proteins and suggest that the NF90ctv isoform (lacking the RGD and GQSY domains) has tenfold higher dsRNA binding properties. In addition we find that NF90ctv interacts with considerable specificity with regions of the structured HIV-1 TAR RNA domain. This was demonstrated by Northwestern blotting and competition assays using TAR-RNA mutants. Biological effects of NF90ctv, we reasoned, may in part be due to its affinity for the HIV-1 TAR element which results in disruption of specific steps in viral replication cycle that depend upon TAR RNA-host protein interaction.

Transcription activation by Tat occurs through TAR and requires proper folding of the TAR RNA hairpin structure [49]. The role of TAR in regulating HIV-1 gene expression has been extensively investigated by using *in vitro *transcription, transient expression analysis and nuclear run-on experiments [50-53]. Binding of HIV-1 Tat protein to the TAR RNA structure is critically dependent upon the positive transcription elongation factor b (P-TEFb), composed of cyclin-dependent kinase 9 (CDK9) and cyclin T1. We reported that the Tat/TAR-dependent P-TEFb kinase activity is required for phosphorylation at Ser 2 and Ser 5 of RNAPII C-terminal domain repeats. Importantly, inhibition of the P-TEFb kinase activity reduced methylation of histone H3 lysine 4 in integrated HIV-1 chromatin [12]. These studies demonstrate that the TAR RNA loop, bulge, and stem structure are each critical for viral gene expression. These unique features of the TAR secondary structure encode information whose downstream signaling is vital for viral replication. We envisaged that NF90ctv recognition of the TAR region may provide a potential barrier in recruitment of Tat and associated cellular factors that results in down regulation of viral gene transactivation.

We approached the issue by biochemical binding/competition assays, transcription *in vivo *as well as by examining the epigenetic modifications of HIV-1 genome to assess the consequence of competing Tat/TAR RNA interaction in the presence of NF90ctv. We utilized pHIV-1 LTR-β gal and pSV-tat constructs to transfect HeLa cells in the presence and absence of NF90ctv. The basal level of transcription of HIV-1 LTR as judged by β-galactosidase reporter gene expression was stimulated by Tat. However, addition of NF90ctv significantly inhibited Tat-mediated transactivation. These results suggest that NF90ctv may effectively sequester TAR RNA and block Tat-TAR interaction thereby limiting Tat-mediated transactivation of HIV-1 transcription. Importantly, the competition of Tat-TAR RNA interaction mediated by NF90ctv leads to epigenetic marks of transcriptionally silenced HIV-1 chromatin.

Inhibition of the Tat-TAR interaction is considered as a realistic approach to develop new anti-HIV compounds. Here we present a novel HIV-1 Tat/TAR antagonist, NF90ctv which inhibits Tat transactivation and attenuates viral production. Examining host cell response as a result of constitutive expression of NF90ctv, Krasnoselskaya-Riz et al, [30] reported that NF90ctv expressing cells induce IFN-response genes which in part accounts for the HIV-1 resistance.

In summary, we have identified a novel cellular protein that is able to bind to the TAR element and suppress Tat-mediated transcriptional trans-activation of HIV-1 LTR. NF90ctv recognition of TAR enables it to inhibit specific steps in the viral life cycle, in parallel with the activation of innate antiviral defenses of the host.
